# Adolescents’ Engagement With an mHealth Multiple Health Behavior Change Intervention (LIFE4YOUth): Mixed Methods and Qualitative Comparative Analysis

**DOI:** 10.2196/88054

**Published:** 2026-03-12

**Authors:** Anna Seiterö, Pontus Henriksson, Kristin Thomas, Marie Löf, Marcus Bendtsen, Ulrika Müssener

**Affiliations:** 1 Department of Health, Medicine, and Caring Sciences Linköping University Linköping, Östergötland Sweden; 2 Department of Medicine Huddinge Karolinska Institutet Stockholm Sweden

**Keywords:** mHealth, adolescents, health behaviors, user engagement, mixed methods, qualitative comparative analysis

## Abstract

**Background:**

Behavior change interventions delivered through mobile phones often have low engagement among end users.

**Objective:**

This study aimed to explore factors influencing engagement among Swedish high school students with access to LIFE4YOUth, a mobile-based multiple behavior change intervention targeting physical activity, diet, alcohol consumption, and cigarette smoking. Special emphasis was placed on understanding low engagement.

**Methods:**

A sequential explanatory mixed methods design was used. Quantitative usage data from 377 students were analyzed to describe engagement patterns. This was followed by qualitative data collection through 3 focus groups and 2 individual interviews (n=20), analyzed using inductive content analysis. Finally, qualitative comparative analysis (QCA) was used to integrate findings and identify configurations of psychosocial and behavioral conditions associated with low engagement. The results from all phases were interpreted and discussed as a whole.

**Results:**

A majority (253/377, 67%) of participants showed low engagement, with 62% (158/253) never interacting with the intervention beyond receiving weekly SMS text messaging. Focus group discussions revealed 3 overarching categories influencing engagement: perceived importance of behavior change, user experiences, and environment of use. In total, 48% (121/253) of the low-engaged participants were represented by 1 of 3 configurations, which described participants’ characteristics as unmotivated high-needers, motivated low-needers, and dissatisfied needers. Robustness tests confirmed the stability of the unmotivated high-needers configuration.

**Conclusions:**

LIFE4YOUth (Linköping University) did not engage high school students with multiple risk behaviors who were content with their lives and did not consider healthy behaviors as very important. However, positive experiences of being both confirmed and encouraged may explain engagement among students engaged in a combination of health-risk and health-promoting behaviors. Future research could explore how tailoring the number of behaviors targeted by mHealth interventions for adolescents might increase engagement and, in turn, behavioral outcomes.

## Introduction

Health-risk behaviors among adolescents, such as insufficient physical activity, poor diet, heavy episodic drinking, and cigarette smoking, call for effective strategies to increase adolescents’ engagement in healthy behaviors [1-3]. Research shows that interventions that use mobile health (mHealth) technology have the potential to influence adolescents’ health behaviors [4-6]. This finding is promising because it implies that many adolescents can be reached concurrently at low costs regardless of geographical location [7].

Despite the potential of mHealth to reach adolescents anytime and anywhere while preserving their integrity in relation to peers and professionals [8], evidence indicates that it is often challenging to engage adolescents with mHealth tools [9,10]. The mHealth engagement typically includes behavioral, cognitive, and emotional aspects of the interaction between the user (eg, adolescent) and the technical platform, such as a mobile phone health app. Although these dimensions of engagement interact [11], the behavioral aspect has received the most attention in the scientific literature to date [12,13]. For example, studies that rely on usage data combined with user demographic data to investigate factors associated with, and potentially predictive of, intervention usage in general [14] and effective engagement in particular [10,15,16].

However, aside from the limited number of empirical studies that seek to understand and explain engagement patterns from broader perspectives, including cognitive and emotional aspects of engagement, several important knowledge gaps remain. First, since the use of multiple behavior change approaches to tackle the co-occurrence of health risk behaviors is still in its infancy, there is limited knowledge about user engagement with mHealth interventions that target multiple behaviors simultaneously [17,18]. Second, the existing evidence on contextual factors associated with mHealth engagement is primarily based on adult populations [17,19,20], underscoring the need for studies focusing specifically on adolescents’ engagement with mHealth behavior change interventions [13]. To address these gaps, studies have been conducted to examine design features associated with sustained engagement [21,22] and to identify effective strategies for increasing engagement among adolescent users of mHealth behavior change tools [23]. However, knowledge about what influences and explains engagement patterns among adolescents with access to an mHealth multiple behavior change intervention is still limited.

Consequently, this mixed methods study aimed to investigate explanations for engagement among high school students with access to an mHealth multiple behavior change intervention (LIFE4YOUth; Linköping University), with a specific emphasis on understanding low engagement. The following research questions (RQs) were examined:

- RQ1: What characterizes high school students’ engagement with the LIFE4YOUth intervention during a 16-week intervention period?
- RQ2: What conditions are plausible explanations for high school students’ engagement with the LIFE4YOUth intervention?
- RQ3: Which configurations of psychosocial and behavioral conditions may explain low engagement?

## Methods

### Study Design

This study was part of a project that overall aimed to evaluate the effects of a multiple behavior change mHealth intervention (LIFE4YOUth) targeting Swedish high school students [24]. A sequential 3-phased explanatory mixed methods design [25] with qualitative comparative analysis (QCA) [26] was used to understand engagement patterns. Integration occurred at each stage of the study. In phase 1 (RQ1), baseline and usage data were analyzed using descriptive statistics to give an overall idea of how high school students engaged with LIFE4YOUth during a 16-week intervention period. Results from Phase 1 were used to develop an interview guide and diagrams depicting engagement patterns for the sequential phase. In phase 2 (RQ2), high school students participating in focus groups were encouraged to interpret the depicted engagement patterns through discussion and reflection based on their experiences with LIFE4YOUth. An inductive approach of qualitative content analysis was used for analyzing the qualitative data [27]. Explanations for low engagement discussed among focus group participants were thereafter used to conceptualize and build the conditions that were further investigated in Phase 3. In phase 3 (RQ3), conditions revealed from phase 2 were systematically compared using QCA to identify combinations of conditions (ie, configurations) that specifically occurred among low-engaged participants. In line with the sequential explanatory mixed methods design, the qualitative and quantitative results were ultimately integrated and interpreted together to provide a comprehensive understanding of explanations for engagement among high school students with access to an mHealth multiple behavior change intervention (LIFE4YOUth). All procedures are reported in accordance with the COREQ (Consolidated Criteria for Reporting Qualitative Research) 32-item checklist [28] and the Good Reporting of A Mixed Methods Study (GRAMMS) checklist [29].

### LIFE4YOUth Intervention

The LIFE4YOUth intervention was developed as part of the Mobile health Multiple lifestyle Behaviors Interventions across the Lifespan (MoBILE) research program, which designs and evaluates mHealth interventions tailored for different populations [30]. LIFE4YOUth was designed to promote healthy behaviors among Swedish high school students. Three intervention components were central: (1) a weekly screening and feedback feature; (2) a dashboard with factual information and exercises for all health behaviors; and (3) an SMS text messaging service that can be activated by end users that includes automatic SMS text messaging programs for each health behavior (physical activity, diet, alcohol consumption, and cigarette smoking), as described in detail elsewhere [24,31]. All content was tailored to the target group [32] and based on recommendations provided by the National Board of Health and Welfare in Sweden [33], previous studies [34,35], and health behavior theories [36,37]. The LIFE4YOUth trial indicated a potential of LIFE4YOUth to increase participants’ weekly time spent in moderate to vigorous physical activity after 2 and 4 months and to a small extent increase their short-term intake of fruit and vegetables. The effects on alcohol consumption and cigarette smoking were less convincing [31].

### Phase 1: Characterizing Engagement Patterns (RQ1)

#### Data Collection

Quantitative data were collected from participants (n=377) in the intervention arm of the LIFE4YOUth trial [31]. The trial participants were recruited from high schools across Sweden, including vocational training and education preparing students for higher studies. The eligibility criteria were being a high school student, not meeting health recommendations for either physical activity, diet, alcohol consumption, or cigarette smoking, and owning a mobile phone. No exclusion criteria were applied. A total of 1398 students registered interest in the study, of whom 890 (64%) consented to participate, and 760 completed the baseline questionnaire, which was used to assess eligibility. Four (0.5%) students did not meet the eligibility criteria and were therefore excluded. All participants were enrolled in the trial between September 2020 and June 2023.

#### Engagement Measures

Three engagement measures were used to describe participants’ engagement with LIFE4YOUth: (1) activation of the SMS text messaging program to support behavior change (yes or no), (2) the number of weeks completing the weekly screening assessment (1-16), and (3) the total number of sessions interacting with the content, defined as a continuous series of screen views initiated by actively clicking on symbols to navigate between dashboard content (zero to infinity). A new screen view after 30 minutes of inactivity was considered a new session [38].

#### Explanatory Measures

Explanatory measures referred to baseline variables that were assumed to influence participants’ engagement with the intervention. These included 4 psychosocial and 7 health behavioral variables. The choice of psychosocial variables was based on common constructs in behavior change theories [36,37] assumed to mediate the effects of LIFE4YOUth [39]. Importance, confidence, and know-how were generally measured using the following questions: (1) “How important do you think it is to improve your lifestyle or sustain your healthy behaviors?”; (2) “How confident are you that you will be able to change your lifestyle or sustain your healthy behaviors?”; (3) “How well do you know how to change your health behaviors?” Responses were given on a 10-point Likert scale. As adolescents’ satisfaction with life is also associated with their health behaviors [40,41], life satisfaction was also included as a psychosocial variable; and assumed to influence engagement. Life satisfaction was measured using Cantril’s ladder [42]: (4) “Thinking about your own life and personal circumstances, how satisfied are you with your life as a whole?” Responses were recorded on an 11-point Likert scale ranging from 0 (not at all satisfied) to 10 (completely satisfied).

The behavioral variables assumed to influence engagement included (1) weekly time (minutes) spent in moderate to vigorous physical activity (MVPA); (2) number of daily portions (100 g) of fruit and vegetables; (3) number of weekly portions of candy or snacks; (4) number of weekly sugary drinks (33 cL) consumed; (5) monthly frequency of heavy episodic drinking (ie, ≥ 4 standard drinks at 12 g pure alcohol on one occasion); (6) number of weekly standard drinks of alcohol consumed; and (7) smoking any cigarette in the past week (yes or no). All variables except for smoking were measured numerically through self-reported data, as described in detail elsewhere [31].

#### Data Analysis

The k-means function in R (version 1.3.1073; R Core Team) was used to create 3 engagement clusters (high, medium, and low) based on participants’ (1) activation of text-message programs, (2) the number of weekly screening assessments, and (3) the number of sessions interacting with the dashboard content. Two-sample *t* tests and chi-square tests were used to test for statistically significant differences between clusters with respect to baseline characteristics, using a significance level of 0.05. The k-means clustering was also used to identify crossover points for all psychosocial variables included in the QCA.

### Phase 2: Identifying Conditions Explaining Engagement Patterns (RQ2)

#### Data Collection

High school students participating in the LIFE4YOUth trial [31] were invited to participate in a focus group interview. Two high schools in the region of Östergötland invited students to focus groups using a convenient sampling strategy [27]. The schools were chosen based on their different educational profiles and the fact that contact with staff was established. Students were recruited either by written information provided by the school nurse or by oral information from teachers. All students in 2 classes were invited to participate during school hours. Three students within these classes declined participation. A total of 3 focus groups and 2 individual interviews with a total of 20 high school students were conducted by AS (a female PhD candidate and experienced school nurse) and UM (a female senior associate professor with expertise in qualitative methodology). No one other than the students and moderators, who were not familiar with the participants, attended the focus groups.

Both interviews and focus groups, which involved 4-8 participants, were audio-recorded and conducted at the participants’ school during school hours between February 2023 and April 2023. A semistructured interview guide with predefined themes was used to facilitate the focus groups. The discussions were initiated by showing participants diagrams illustrating engagement patterns (derived from Phase 1) and asking them to reflect on the factors that might explain each pattern based on their experiences with LIFE4YOUth. The duration of the sessions varied between 33 and 46 (mean 40, SD 6) minutes. Field notes were written directly after each session.

#### Data Analysis

The audio-recorded data were transcribed verbatim by professionals and analyzed inductively using qualitative content analysis [27]. All transcripts were carefully read by AS and UM and checked for accuracy against the audio recordings (AS). The coding procedure was performed using the software (NVivo 12 Plus; QSR International Pty Ltd) to organize similar content in the data using preliminary labels (ie, codes; AS). After establishing a coding scheme, all transcripts were reread to systematically code all relevant text units (AS). Text extracts for each code were read and reread to get an idea of similarities and differences among data within codes and between codes. Codes with similar content were combined to create categories, which were checked against the transcripts to verify meaningfulness and to ensure that all relevant data were captured by the categories. UM was involved in all analytical procedures through repeated discussions until consensus was reached.

### Phase 3: Identifying Combinations of Conditions Explaining Low Engagement (RQ3)

#### Building Integration Using QCA

A crisp set QCA [26,43] was conducted to explore whether the conditions derived from the qualitative findings helped explain the low-engagement cluster identified in Phase 1. Crisp sets were used because of our interest in exploring whether the presence rather than the degree (ie, fuzzy set) of a condition was related to engagement. While the qualitative findings from Phase 2 informed the selection and definition of conditions, k-means clustering was used to determine the crossover points used to calibrate each condition. All participants at or above the crossover point were coded as 1 (fully in the set), while cases below the crossover point were coded as 0 (fully out). For negatively framed conditions, the calibration was reversed: cases at or below the crossover point were coded as 1, and cases above the crossover point were coded as 0. A truth table [26,44] was constructed to obtain an overview of all possible combinations of conditions (ie, configurations) and their coverage in empirical data. The analysis also included configurations represented by only one participant, which is coherent with good practice of QCA [45,46]. An enhanced standard analysis [47] was carried out by logically minimizing the truth table. A consistency threshold at 0.75 was deemed enough to reveal descriptive patterns in the data. The minimization procedure, as with all other QCA procedures, was performed using the QCA packages (version 3.19) and SetMethods (version 4.0) in R (version 1.3.1073). The algorithm used to minimize the truth table starts at the upper row and systematically works through each truth table row with an outcome value of 1 to determine whether a condition can be eliminated from the configuration. The goal of this procedure is to find the briefest way to summarize all available information from the truth table, that is, the most parsimonious solution [26]. All configurations included in the most parsimonious solution indicate alternative pathways to low engagement. All configurations were interpreted holistically based on knowledge gained from the qualitative phase and were labeled to describe the overall characteristics conveyed by each pathway.

#### Robustness Tests

The analytical procedures were repeated using the negation of the outcome (not low engagement), in accordance with recommendations [45,46]. The model analytics included interpretation of consistency and coverage, evaluating the simplifying assumptions used in the logical minimization (ie, whether possible configurations that were not represented by data were used in the minimization), and assessing model ambiguity (ie, whether the minimization revealed more than one valid way of summarizing the truth table).

Following Oana and Schneider’s approach [48], robustness was further assessed using a fit-oriented and case-oriented approach to measure how sensitive the solution was to analytical decisions. The obtained solution was tested against alternative solutions derived from the following analytical decisions using a consistency threshold of 0.8 instead of 0.75, not excluding participants with activated SMS text messaging programs from the low engagement outcome, and using 5 as a frequency threshold (ie, including configurations represented by at least 5 participants in the minimization of the truth table). The obtained solution was compared to the minimal test solution, which is the area where all possible solutions agree, and the maximal test solution, which is the entire area of possible solutions. This comparison was made to determine how much the 2 sets of solutions overlapped. Robustness was also assessed in terms of the proportion of participants that would remain in the alternative solution.

### Ethical Considerations

Students were informed about the LIFE4YOUth intervention through printed advertisements distributed in high school settings. Before providing consent to participate in the study, all participants received written and oral information that briefly described the characteristics of the 16-week digital intervention, the purpose and design of the study, the types of data collected, how data will be handled, and that participation is voluntary and without any financial compensation. All participants were further informed that they were free to decide if, how, and when to use the digital intervention, and that they had the right to withdraw from the study at any point without consequences. When applicable, participants were informed about the role of the focus group moderators as part of the research team. Informed consent was collected from all participants (aged 15 years or older) but not from their guardians. All quantitative data were pseudoanonymized using a user identity, which only one co-author (MB) had access to. To further ensure privacy and confidentiality, only those involved in the collection and analysis of focus group data (AS and UM) had access to personal information linked to the data. The procedures to collect and use quantitative engagement data (Swedish Ethical Review Authority, Dnr 2019-03813, Dnr 2020-03538) and qualitative data (Dnr 2022-02493-02) were consistent with the ethical approval from the Regional Ethical Committee in Linköping, Sweden. No financial compensation was offered for participation.

## Results

### Overview

The characteristics of the study sample are presented in Table 1. The median age of participants was 17 years. Females were overrepresented (265/377, 70%).

**Table 1:** Overview of participants specified per dataset and engagement pattern.

Participant characteristics			Qualitative (n=20)			Quantitative engagement (n=377)							
						Total (n=377)		Low (n=253)		Medium or high (n=124)		*P* value^a^	
Age (years), mean (SD)			17-19^b^			17.18 (1.20)		17.19 (1.19)		17.15 (1.23)		.81	
**Sex, n (%)**													.11
	Female	15 (75)			265 (70)		179 (71)		86 (69)				
	Male	5 (25)			112 (30)		74 (29)		38 (31)				
**Parents’ education^c^** **, n (%)**													.58
	Primary	—^d^			25 (7)		17 (7)		8 (6)				
	Secondary	—			131 (35)		90 (36)		41 (33)				
	Tertiary	—			221 (59)		146 (56)		75 (60)				
**Economy^e^** **, n (%)**													.29
	Very good	—			112 (30)		76 (30)		36 (29)				
	Average	—			232 (62)		158 (62)		74 (60)				
	Not so good	—			30 (8)		16 (6)		14 (11)				
	Not good at all	—			3 (1)		3 (1)		0 (0)				
**Region of birth^f^** **, n (%)**													.81
	Sweden	—			324 (86)		219 (87)		105 (85)				
	Other	—			53 (14)		34 (13)		19 (15)				
**Parents’ region of birth^f^** **, n (%)**													.31
	Sweden	—			268 (71)		181 (72)		87 (70)				
	Other	—			109 (29)		72 (28)		37 (30)				
Satisfaction with life^g^, mean (SD)						6.83 (1.99)		6.91 (1.98)		6.67 (2.03)		.28	
**Psychosocial variables^h^** **, mean (SD)**													
	Importance	—			6.66 (2.53)		6.45 (2.58)		7.07 (2.39)		.03		
	Knowledge	—			6.65 (2.31)		6.99 (2.32)		6.59 (2.27)		.05		
	Confidence	—			6.24 (2.52)		6.17 (2.54)		6.38 (2.48)		.45		
**Behavioral variables, mean (SD)**													
	Weekly time spent in MVPA^i^ (minutes)			—	323 (316)		321(310)		326 (329)		.89		
	Number of daily portions (100 g) of fruit and vegetables consumed			—	1.43 (1.14)		1.37 (1.08)		1.56 (1.26)		.14		
	Number of weekly sugary drinks (33 cL) consumed			—	3.27 (4.45)		3.53 (4.75)		2.75 (3.73)		.11		
	Number of weekly portions of candy or snacks consumed			—	4.60 (5.55)		4.29 (5.19)		5.24 (6.19)		.12		
	Monthly frequency of heavy episodic drinking (ie, ≥4 standard drinks)			—	1.21 (2.86)		1.47 (3.35)		0.69 (1.22)		.01		
	Number of weekly standard drinks (12 g pure alcohol) consumed			—	1.75 (4.10)		2.03 (4.49)		1.18 (3.11)		.06		
	Smoking any cigarette in the past week, n (%)			—	68 (18)		53 (21)		15 (12)		.05		

^a^Based on 2 sample *t* tests of means and chi-square tests for proportions.

^b^Range for the qualitative dataset.

^c^Assessed as “Please select the highest education for your mother and father.”

^d^Not available.

^e^Assessed as “How would you describe the economic situation in your family?”

^f^Assessed as “Where were you/your parents born?”

^g^Cantril’s ladder: “Thinking about your own life and personal circumstances, how satisfied are you with your life as a whole?” Responses were given on an 11-point Likert scale where 0 represents not at all satisfied.

^h^Defined as follows: Importance: “How important do you think it is to improve your lifestyle or sustain your healthy behaviors?” Confidence: “How confident are you that you will be able to change your lifestyle or sustain your healthy behaviors?” Knowledge: “How well do you know how to change your health behaviors?” Responses were provided on a 10-point Likert scale.

^i^MVPA: moderate to vigorous physical activity.

The majority were born in Sweden (324/377, 86%) with highly educated Swedish-born parents. An overview of participants’ baseline characteristics across all engagement levels is provided in Table S1 in Multimedia Appendix 1.

### Phase 1: High School Students’ Engagement With LIFE4YOUth (RQ1)

#### Defining Engagement Patterns

The results from this phase describe the characteristics of participants’ engagement with the LIFE4YOUth intervention. The results from the k-means clustering (Table S2 in Multimedia Appendix 1) informed the thresholds used to define 3 engagement patterns. Low engagement was defined as 2 or fewer weeks (out of 16) of completing the weekly screenings, a maximum of 3 sessions of engagement with the dashboard content, and no activated SMS text messaging program. High engagement was defined as completing the weekly screening in at least 9 weeks, or by 12 or more engagement sessions during the intervention period, or by having activated at least one SMS text messaging program. Engagement between low and high was defined as a medium level of engagement.

#### Engagement With the LIFE4YOUth Intervention

Among participants who engaged with LIFE4YOUth at least once (219/377, 58%) during the 16-week intervention period, the weekly screening was completed on average in 4 (SD 4) weeks, and the dashboard content was accessed on average 5 (SD 5.5) sessions. The proportion of participants who completed the weekly screening assessment was almost identical to the proportion per week who engaged with the dashboard content. Twenty-one percent of participants (45/219) who engaged at least once activated one or more SMS text messaging programs focusing on physical activity, diet, alcohol consumption, or cigarette smoking. Table 2 presents an overview of engagement activities by engagement cluster.

**Figure 1:**
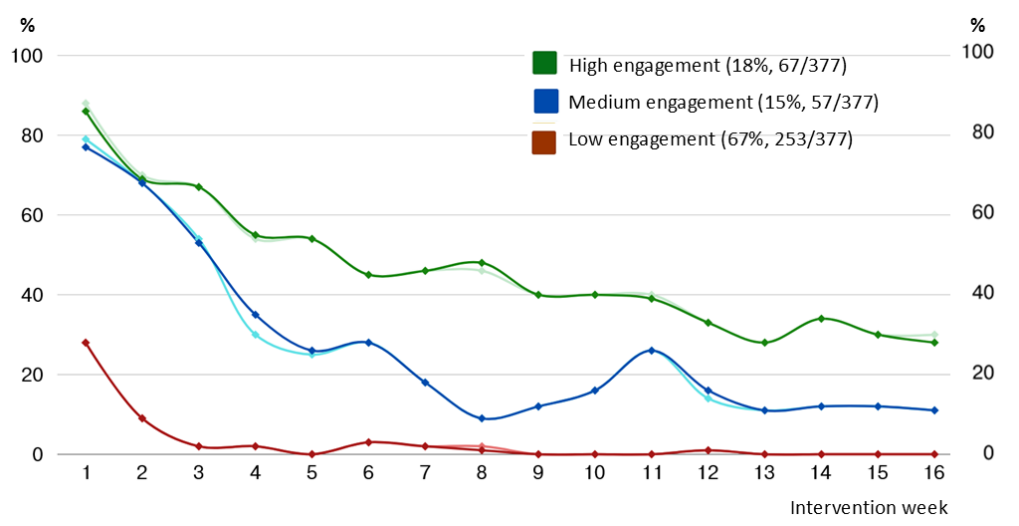
Proportion of participants who completed the weekly screening (darker color) and engaged with the dashboard content (lighter color), by engagement cluster and intervention week.

In total, 18% (67/377) were defined as high-engaged, 15% (57/377) were defined as medium-engaged, and 67% (253/377) were defined as low-engaged (Figure 1). Sixty-two percent (158/253) of the low-engaged participants never engaged actively. At baseline, low-engaged participants reported significantly higher frequency of heavy episodic drinking, lower levels of perceived importance, and higher levels of knowledge of how to change their behaviors. These differences were investigated further in the subsequent phases to gain a better understanding of what explains engagement in general (Phase 2) and low engagement in particular (Phase 3). Engagement patterns were similar throughout the study period, with no statistically significant differences during or after the COVID-19 pandemic (Table S3 in Multimedia Appendix 1).

**Table 2:** Cluster-specific engagement measures.

Engagement measures	Low (n=253)	Medium (n=57)	High (n=67)
SMS text messaging programs, n (%)	0 (0)	0 (0)	45 (67)
Weekly screenings, mean (SD)	0.49 (0.69)	4.25 (1.66)	7.46 (5.28)
Session episodes, mean (SD)	0.54 (0.79)	5.35 (2.37)	9.75 (7.40)

### Phase 2: Conditions Explaining High School Students’ Engagement With LIFE4YOUth (RQ2)

#### Categories of Conditions That Overall Influence Engagement

The results from this phase describe conditions identified among a subset of participants (n=20) from phase 1 who took part in focus groups to provide insights and help interpret the previously identified engagement patterns. Three categories of plausible explanations for engagement with LIFE4YOUth were discussed among focus group participants: (1) perceived importance of health behavior change, (2) user experiences, and (3) environment of use. Descriptions of these categories are followed by suggestions regarding how these conditions were perceived to explain medium and high engagement as well as low engagement.

#### Perceived Importance of Health Behavior Change

Focus group participants considered the perceived importance of healthy behaviors as a product based on their interest, willingness, and intention to act to accomplish change. Experiences of problems, such as being dissatisfied with life or oneself, were considered reasons for being interested in behavior change. However, the components of willingness and intention were perceived to be influenced by students’ beliefs about their ability to cope with the required efforts given the current circumstances in their lives. Thus, the perceived importance of health behavior change as a condition for engagement incorporates confidence in behavior change. Indeed, participants stressed that one can be both interested and have a desire to change something but lack the confidence to make changes, a constraint that impacts perceptions about the importance of a behavior change, as illustrated in the quotations below:

When one isn’t doing well, one may easily think of oneself as incapable and avoid trying to establish new habits because being too busy thinking about other things and doesn’t want any changes when one is at least somewhat happy.

Female

It’s all about your attitude to change behaviors, [. . .] to notice the tips and acknowledge what they might do for you, you need to have a desire to be helped.

Male

Well, the first two times I used it [LIFE4YOUth], I browsed and read. After a while, I realized that there’s nothing I want to actively change right now.

Female

#### User Experiences

The discussions among participants revealed that the experience of gaining something expected from the intervention—for example, gaining knowledge and adequate support—is critical for an overall positive user experience. The conditions the user experiences further include reactions elicited by the intervention, such as positive emotions of inspiration or negative emotions of defense. Emotions evoked can vary depending on current behaviors and social values. Indeed, some participants perceived that even study content that was not part of the intervention (eg, baseline weight question) elicited negative emotions:

It [study content] can be a reminder to always have to think about it, and when life struggles on and you know that ‘yeah, I am aware about what I am doing may not be the best, but I don’t have time to think about it’.

Female

Participant 1: I think that people always seek validation [. . .], it doesn’t have to be something material, but as you said about feedback . . . Receiving feedback, such as grades or whatever, [. . .] is crucial, because if you do all the things and get nothing in return, then you will probably not . . . Participant 2: I also think that you want to gain something from it [the intervention]. Such as knowledge or, as you said, validation, and insights on how to improve oneself.

Male

#### Environment of Use

The condition environment of use refers to social circumstances that influence when and how high school students engage with LIFE4YOUth. The participants emphasized that many things require high school students’ attention that compete with how much time they spend with LIFE4YOUth. In fact, participants argued that it may be merely a lucky coincidence if a LIFE4YOUth SMS text messaging is received at a suitable time, which may slightly increase the likelihood of engaging with the intervention by clicking on the link in the SMS text messaging. On the other hand, receiving the SMS text messaging while busy means that the message will likely be forgotten:

Depending on when it is received [the LIFE4YOUth text message], one has a varied amount of time to actually respond to the questions. If I was studying at home and received a [LIFE4YOUth] text message, [. . .] I would likely take a break. But if you’re with friends and get a text-message, it’s not like, ‘Yeah, chill, I should just respond to some questions.’

Male

There are other [mobile-phone health] apps that only aims at smoking cessation [. . .], but this app [LIFE4YOUth] should aid . . . it serves several purposes. However, the other app may only have one function, and that is to make you stop smoking.

Female

#### Suggested Explanations for Medium or High Engagement With LIFE4YOUth

Participants reasoned that when one is satisfied with life, one tends to be happier, more confident, curious, and interested in improving oneself. Satisfied people who are engaged in some behaviors considered healthy and in some behaviors considered risky can experience that LIFE4YOUth confirms their healthy behaviors and encourages them to avoid their risky behaviors. The experience of being validated and encouraged can spark ambitions and increase the likelihood of end users continuing to be engaged with LIFE4YOUth. Thus, positive reactions were considered a mechanism that explains engagement. Furthermore, the content provides tips for the adaptation of health-promoting behaviors. According to the participants, when experiencing the tips as desirable and helpful based on a learning need, end users may engage with LIFE4YOUth to a greater extent, indicating that meeting expectations also constitutes a mechanism that drives engagement.

Furthermore, participants thought that end users who are not very happy with their lives and want a change to feel better might consider LIFE4YOUth as a tool worth trying, even when not being convinced that a mobile phone app will help them. Under such circumstances, expectations regarding one’s ability to improve health behaviors and the perceived helpfulness of LIFE4YOUth were lower than when the goal was to successfully change those behaviors. That is, the influence of confidence was perceived to be less meaningful in such situations.

#### Suggested Explanations for Low Engagement With LIFE4YOUth

Participants emphasized that end users with several health-risk behaviors are unlikely to want to be reminded of this on a regular basis. This was deemed to be stressful, that is, a negative reaction to LIFE4YOUth. In addition, participants believed that one may not agree with indicators about health behaviors being considered a health risk. Some participants underlined that behaviors vary from week to week and that their behaviors overall might be adequate. Furthermore, LIFE4YOUth addresses multiple health behaviors, and end users with limited knowledge, or who seek in-depth knowledge about a specific behavior, may find the content too broad. According to participants’ reasoning, the risk of being disappointed increases as end users’ expectations or needs increase. Higher needs can, for example, depend on current health behaviors or on overall circumstances that impact end users’ capabilities to manage their situation.

Two additional explanations for low engagement were raised during the discussions. It was not assumed that end users experiencing life challenges possessed the necessary energy or interest to engage in behavior change. In fact, participants generally found it far too easy to disengage because no one pursued them or even encouraged them, as parents might with their schoolwork. When being satisfied with life, on the other hand, one may have no desire to change anything, and engagement with LIFE4YOUth might not be considered as worthwhile. Furthermore, the weekly SMS text messaging itself served as a reminder about health behaviors. Some participants did not believe that active engagement was therefore needed to benefit from the intervention.

### Phase 3: Combinations of Conditions Explaining Low Engagement (RQ3)

#### Defining Conditions for the QCA

The integrated results from this phase describe how the conditions identified in phase 2 may help explain low engagement within the phase 1 sample (n=377). Based on the discussions among focus group participants, 5 conditions were selected, defined, and included in the QCA. These were (1) engagement in health-risk behaviors; (2) engagement in health-promoting behaviors; (3) being dissatisfied with life; (4) being high-skilled in how to change behaviors; and (5) considering healthy behaviors as very important.

Engagement in health-risk behaviors was defined as smoking ≥1 cigarette in the past week, or (if ≤18 years old) drinking any alcohol in the past week, or (if ≥18 years old) having consumed ≥10 standard drinks of alcohol in the past week, or engaging in heavy episodic drinking ≥1 time in the past month. Health-promoting behaviors were defined as ≥420 minutes of MVPA in the past week or ≥3 daily portions of fruit and vegetables in the past week, combined with either ≤4 portions of candy and cakes in the past week or ≤2 cans (33 cL) of sugary drinks in the past week. These cut-off points align with the thresholds used in the screening and feedback component of LIFE4YOUth. Dissatisfaction with life was defined based on the results of the k-means clustering (≤5 on a 11-point Likert scale). Being highly skilled in how to change health behaviors, as well as considering healthy behaviors as very important, was defined as ≥8 on a 10-point Likert scale based on the k-means clustering.

#### Findings From the QCA

Table 3 shows an overview of all 32 possible combinations of conditions (ie, configurations) and their representation among participants in this dataset. The minimization of these configurations and their empirical representation assisted in identifying 3 configurations that contribute to explaining low engagement. No simplifying assumptions were applied to obtain the most parsimonious solution, and no model ambiguity occurred, meaning that none of the configurations associated with low engagement were also associated with non–low-engagement. The most parsimonious solution model was identical to the conservative and intermediate solution models, indicating a high degree of robustness in the identified configurations. The robustness test identified one configuration that remained stable across all tested changes, including participants who were not engaged in health-promoting behaviors but were engaged in health-risk behaviors, did not consider it important to change their health behaviors, and lacked knowledge of how to change them were likely to show low engagement with LIFE4YOUth. Details of the robustness tests are provided in Tables S4 and S5 in Multimedia Appendix 1.

The 26% raw coverage presented in Table 4 indicates that 26% (66/253) of all low-engaged participants demonstrated the configuration labeled unmotivated high needers. Within this configuration, participants engaged in health-risk behaviors but not in health-promoting behaviors, were not dissatisfied with their lives, and did not consider healthy behaviors to be very important. The consistency value of 0.83 means that, of all participants who were described by this configuration, 83% (69/80) were low-engaged. The configurations motivated low needers and dissatisfied needers to describe 2 additional pathways to low engagement. The first pathway reflects being engaged in health-promoting behaviors, considering healthy behaviors to be very important, and not being dissatisfied with life. The second pathway reflects being engaged in health-risk behaviors, not being very skilled in how to change behaviors, and being dissatisfied with life. In total, 48% (121/253) of all low-engaged participants in this dataset were described by one of these configurations, while 19% (29/151) of those described by the configurations were not low-engaged.

**Table 3:** Truth table, logically possible combinations of conditions in relation to the outcome.

LS^a^	HPB^b^	HRB^c^	IMP^d^	KNOW^e^	OUT^f^	n (%)	Incl^g^
1	1	1	1	0	1	1 (0)	1.000
1	0	1	0	0	1	11 (4)	0.909
0	0	1	0	0	1	57 (23)	0.842
0	1	1	1	1	1	5 (2)	0.800
0	0	1	0	1	1	23 (9)	0.783
0	1	0	1	1	1	13 (5)	0.769
1	0	1	1	0	1	13 (5)	0.769
0	1	0	1	0	1	16 (6)	0.750
0	1	1	1	0	1	8 (3)	0.750
1	1	1	0	0	1	4 (2)	0.750
0	0	1	1	0	0	15 (6)	0.733
0	0	0	0	0	0	39 (15)	0.692
0	1	1	0	0	0	22 (9)	0.667
1	1	1	1	1	0	6 (2)	0.667
0	1	0	0	1	0	8 (3)	0.625
1	0	0	0	0	0	15 (6)	0.600
1	0	0	1	1	0	5 (2)	0.600
1	1	0	0	0	0	5 (2)	0.600
1	0	0	1	0	0	12 (5)	0.583
0	0	1	1	1	0	16 (6)	0.562
0	1	0	0	0	0	9 (4)	0.556
0	0	0	1	1	0	20 (8)	0.550
0	0	0	0	1	0	12 (5)	0.500
0	0	0	1	0	0	24 (9)	0.500
1	0	1	1	1	0	2 (1)	0.500
0	1	1	0	1	0	5 (2)	0.400
1	0	1	0	1	0	3 (1)	0.333
1	0	0	0	1	0	2 (1)	0.000
1	1	0	1	0	0	6 (2)	0.000
1	1	0	0	1	—^h^	0 (0)	—
1	1	0	1	1	—	0 (0)	—
1	1	1	0	1	—	0 (0)	—

^a^LS: being dissatisfied with life (≤5 on a 11-point Likert scale).

^b^HPB: health-promoting behavior (≥420 min MVPA or ≥3 daily portions [100 g] of fruit and vegetables and <4 portions of candy or cakes or <2 cans [33 cL] of sugary drinks).

^c^HRB: health-risk behavior (smoking ≥1 cigarette or being <18 years old and drinking any alcohol or being ≥18 years old and having consumed ≥10 standard drinks [12 g pure alcohol] or being engaged in heavy episodic drinking [≥4 standard drinks on a single occasion] at least once in the past month).

^d^IMP: considering healthy behaviors as very important (≥8 on a 10-point scale).

^e^KNOW: being high-skilled in how to change behaviors (≥8 on a 10-point scale).

^f^OUT: 1=≥75% of all participants within the configuration had low engagement, 0=<75% had low engagement.

^g^Incl: quantifies the degree of a set relationship where 1 indicates a strong relationship between the configuration and the outcome.

^h^Not available.

**Table 4:** The most parsimonious solution contains three configurations that were associated with low engagement with LIFE4YOUth. Overall consistency: 0.81; overall coverage: 0.48.

Configuration^a^	HRB^b^	HPB^c^	LS^d^	KNOW^e^	IMP^f^	Raw^g^	Unique^h^	Consistency
Unmotivated high-needers	Yes	No	No	Yes or No	No	0.26	0.26	0.83
Motivated low-needers	Yes or No	Yes	No	Yes or No	Yes	0.13	0.13	0.76
Dissatisfied needers	Yes	Yes or No	Yes	No	Yes or No	0.10	0.10	0.83

^a^Labeled according to the overall characteristics of the configurations, as interpreted from the findings reported in the qualitative phase.

^b^HRB: health-risk behavior.

^c^HPB: health-promoting behavior.

^d^LS: Being dissatisfied with life.

^e^KNOW: High-skilled in how to change behaviors.

^f^IMP: Considering healthy behaviors as very important.

^g^Raw: the proportion of participants in the outcome set that were covered by the solution term.

^h^Unique: the proportion of participants in the outcome set that are also in the solution term and not covered by any other solution term.

## Discussion

### Principal Findings

This mixed methods study aimed to deepen the understanding of the conditions that influence and explain high school students’ engagement with an mHealth tool (LIFE4YOUth) targeting multiple health risk behaviors (physical activity, diet, alcohol consumption, and cigarette smoking). Overall, the majority of participants who were given access to the LIFE4YOUth intervention engaged to a low extent (Textbox 1). Participants’ perceived importance of behavior change, their experiences with the intervention, and external circumstances that affected their ability to respond to prompts immediately may have influenced their level of engagement. By using participants’ reasoning about explanations for low engagement as a basis for defining conditions in the QCA, the integrated results revealed 3 configurations associated with low engagement. These configurations constitute possible pathways for explaining why adolescents with access to a behavior change tool targeting multiple health-risk behaviors hardly ever interact with the tool. In the discussion that follows, these pathways are interpreted in relation to the findings obtained from the focus groups (phase 2) as well as theory and previous research.

Focus group participants suggested that having multiple risk behaviors—such as insufficient engagement in physical activity and unhealthy dietary behaviors alongside alcohol consumption and cigarette smoking—could explain low engagement among individuals who are content with their lives and do not consider behavior change to be important. This expectation was supported by the quantitative data through the configuration of unmotivated high needers. Indeed, 62% (157/253) of the low-engaged participants disengaged without being exposed to any intervention content other than the weekly SMS text messaging, which raises the question of why so many participants did not interact with the dashboard content even once. It is unlikely that this can be explained by participants’ experiences of the LIFE4YOUth content. However, it is possible that low-engaged participants had no interest in changing their behaviors but enrolled in the trial for other reasons [49]. Therefore, it is possible that the characteristics shared by the low-engaged participants are similar to those of individuals who declined participation in the first place. Low interest in mHealth behavior change interventions can be understood from a novelty-complexity perspective, as suggested by Crutzen and Ruiter [50], who developed a conceptual model on mechanisms that are assumed to influence people’s interest in such tools. People may be interested in behavior change interventions if they perceive the intervention to be novel and the level of complexity of end users’ “problems” is neither too high nor too low. High-level complexity problems are thought to reduce people’s self-efficacy and interest, whereas low-level complexity problems are thought to be simple enough to manage without the assistance of digital tools. Both scenarios can account for low interest in LIFE4YOUth among motivated low-needers, but also unmotivated high-needers and dissatisfied needers who may lack resources relative to their needs. Therefore, it is possible that focusing on multiple behaviors simultaneously within a single intervention became a barrier for some participants to even consider the idea of behavior change.

Although engagement in multiple health risk behaviors and dissatisfaction with life in combination with other explanatory conditions may explain low engagement in some cases, the reverse relationship may also apply. For example, Thornton et al [51] found no evidence of an association between the number of risk behaviors or psychological distress and adolescents’ level of engagement with an app targeting multiple health risk behaviors. This finding is noteworthy, as it resonates with explanations discussed by participants in our focus groups, who suggested that experiencing poor mental well-being might spark interest in using mHealth tools to initiate positive change. This interpretation further aligns with the QCA results for the negated outcome (ie, medium or high engagement; Table S6 in Multimedia Appendix 1), which suggest that dissatisfaction with life, together with nonengagement in health-risk behaviors and other conditions, may be associated with continued engagement in LIFE4YOUth. Thus, adolescents who are struggling in some aspect of their lives may find mHealth lifestyle tools appealing. However, careful tailoring might be needed to empower individuals to believe in their ability to change.

The concept of response efficacy refers to the belief that the “problem” is manageable through the intervention, which is an essential mechanism that can drive continued engagement [50]. Our findings, as well as previous research [9,52,53], show that adolescents who overcome barriers and invest effort in mHealth lifestyle tools may experience these tools as encouraging and remain engaged over time. However, sustained engagement with mHealth tools is not a desired outcome itself [16]. Despite a large proportion of participants in the intervention group of the LIFE4YOUth trial engaging with the content only a few times over the 16-week intervention period, effect estimates [31] indicated positive effects on MVPA after 2 and 4 months and a small 2-month effect on fruit and vegetable intake. Given these effects, the overall level of engagement observed among participants in this study may have been adequate for gaining meaningful insights and reminders that influenced some health behaviors.

Our finding that a large proportion of adolescents engaged with the intervention only a limited number of times further aligns with previous research reporting high levels of nonusage attrition among adolescents using mHealth tools for health promotion [9,52,54]. Peuters et al [9,52] and Maenhout et al [54] explored reasons for engagement and nonengagement with an app targeting physical activity, diet, and sleep among adolescents aged 12-15 years and found that limited interest in behavior change contributed to low engagement. Consequently, it has been suggested that strategies are needed to first increase adolescents’ awareness and motivation to change and thereafter integrate mHealth tools into their everyday lives [54,55].

**Joint display summarizing the results of each phase of this mixed methods study.**

Summary of quantitative, qualitative, and integrated findings.

**Quantitative findings (research question 1 [RQ1])**

A total of 67% (253/377) of participants actively engaged with the intervention content ≤3 times over 16 weeks. Low-engaged participants were significantly more likely to report heavy episodic drinking (mean 1.47, SD 3.35; *P*=.01), lower perceived importance of behavior change (mean 6.45, SD 2.58; *P*=.03), and higher knowledge of how to change their behaviors (mean 6.99, SD 2.32; *P*=.05).

**Qualitative findings (RQ2)**

Three main categories described conditions influencing high school students’ engagement with LIFE4YOUth: (1) perceived importance of health behavior change, (2) user experiences, and (3) environment of use. Low engagement was explained by pressure experienced by participants with poor health behaviors and dissatisfaction with life, or alternatively by high life satisfaction and no desire to change behaviors.

**Integrated findings (RQ3)**

Three configurations associated with low engagement were identified: unmotivated high-needers, motivated low-needers, and dissatisfied needers. These configurations accounted for 48% of all low-engaged participants. Unmeasured circumstances related to user experiences and the environment of usage may explain the remaining cases.

### Implications

Our findings on possible explanations for low engagement as well as medium and higher levels of engagement may help other researchers interpret mHealth engagement data gathered from adolescents. Also, the results presented in this study may be useful in the design of future mHealth interventions aimed at promoting healthy behaviors in adolescents, as they indicate that the multiple behavior approach seemed less feasible for adolescents with the highest need for behavior change. On the other hand, many adolescents are engaged in a combination of health-risk and health-promoting behaviors [56], and our findings suggest that adolescents who experience being both confirmed and encouraged by the intervention may benefit from the multiple-behavior approach. To further examine participants’ engagement with LIFE4YOUth, future research should investigate the quality of adolescents’ engagement with intervention content in more depth, based on self-authored data as well as user-experience data collected from a larger sample of participants. Future research could explore how engagement levels relate to the effectiveness of multiple behavior change interventions in adolescents. It would also be valuable to understand the characteristics of adolescents who are not drawn to digital behavior change support and what types of health-promoting support, if any, they might prefer.

### Strengths and Limitations

This study used a novel design to gain a more nuanced understanding of the conditions and mechanisms that influence and explain high school students’ engagement with a stand-alone mHealth multiple behavior change intervention. The use of a mixed methods design with QCA for integration increased rigor and provided insights beyond those obtained from either quantitative or qualitative data alone, which is considered a strength. There are, however, several important limitations to consider. First, the use of self-reported data on health behaviors increases the risk of recall bias. Importance, confidence, and know-how were additionally measured using a general scale, which limited the ability to capture variability in these constructs across different behaviors [57]. Second, the convenience sampling [58] of the focus groups limited diversity in terms of, for example, socioeconomic status and educational background. The time since focus group participants accessed the intervention also varied, which may have resulted in more speculative statements rather than those based on participants’ experiences. Furthermore, the choice of conditions for the QCA was limited to the data collected in the LIFE4YOUth trial. Several perspectives highlighted by focus group participants, including stress and LIFE4YOUth experiences, were therefore not included in the QCA. Nevertheless, this study was conducted iteratively, with methodological decisions based on the data rather than the reverse.

Finally, all data were collected within the context of a randomized controlled trial, which does not replicate naturalistic circumstances. Biases may have been introduced by the research setting due to research participation effects [59], and it is possible that high school students would have engaged with LIFE4YOUth differently if they had not been part of a research study. We believe, however, that the transparent reporting allows readers to assess whether the findings are valid and generalizable to other contexts.

### Conclusions

Several conditions were identified that may influence and explain high school students’ engagement with the multiple behavior change mHealth intervention (LIFE4YOUth). Low-engaged high school students were more likely to be described as unmotivated high-needers, motivated low-needers, or dissatisfied needers. Emotions and reactions evoked by the intervention, as well as perceptions of whether the intervention aligned with high school students’ support needs, were suggested to explain engagement. In addition, the timing of SMS text messaging prompts was also considered an important condition influencing all levels of engagement. The findings from this study indicate that the multiple-behavior approach was a decisive factor for students with multiple risk behaviors not to remain engaged with LIFE4YOUth, whereas it may have been a better fit for students who felt both confirmed and encouraged by this approach. Future studies can build on these findings to investigate how tailoring the number of behaviors addressed by mHealth tools targeting adolescents may optimize engagement and behavioral outcomes.

## Appendix

Multimedia Appendix 1 Complementary analyses of engagement patterns, including the validity of the identified clusters, the influence of the COVID-19 pandemic on engagement, and the robustness of the results.

## Abbreviations

COREQ: Consolidated Criteria for Reporting Qualitative Research
GRAMMS: Good Reporting of a Mixed Method Study
mHealth: mobile health
MoBILE: Mobile health Multiple lifestyle Behaviors Interventions across the Lifespan
MVPA: moderate to vigorous physical activity
QCA: qualitative comparative analysis
RQ: research question

## Data Availability

The data that support the findings of this study are available from the first author, but restrictions apply to the availability of these data to protect study participant privacy. However, pseudo-anonymized data are available upon reasonable request.

## References

[1] Gore FM, Bloem PJN, Patton GC, Ferguson J, Joseph V, Coffey C, Sawyer SM, Mathers CD (2011). Global burden of disease in young people aged 10-24 years: a systematic analysis. Lancet.

[2] Patton GC, Sawyer SM, Santelli JS, Ross DA, Afifi R, Allen NB, Arora M, Azzopardi P, Baldwin W, Bonell C, Kakuma R, Kennedy E, Mahon J, McGovern T, Mokdad AH, Patel V, Petroni S, Reavley N, Taiwo K, Waldfogel J, Wickremarathne D, Barroso C, Bhutta Z, Fatusi AO, Mattoo A, Diers J, Fang J, Ferguson J, Ssewamala F, Viner RM (2016). Our future: a Lancet commission on adolescent health and wellbeing. Lancet.

[3] Akseer N, Mehta S, Wigle J, Chera R, Brickman ZJ, Al-Gashm S, Sorichetti B, Vandermorris A, Hipgrave DB, Schwalbe N, Bhutta ZA (2020). Non-communicable diseases among adolescents: current status, determinants, interventions and policies. BMC Public Health.

[4] Rodríguez-González P, Iglesias D, Fernandez-Rio J, Gao Z (2023). Effectiveness of interventions using apps to improve physical activity, sedentary behavior and diet: an umbrella review. Complement Ther Clin Pract.

[5] Staiger PK, O'Donnell R, Liknaitzky P, Bush R, Milward J (2020). Mobile apps to reduce tobacco, alcohol, and illicit drug use: systematic review of the first decade. J Med Internet Res.

[6] Talens C, da Quinta N, Adebayo FA, Erkkola M, Heikkilä M, Bargiel-Matusiewicz K, Ziółkowska N, Rioja P, Łyś AE, Santa Cruz E, Meinilä J (2025). Mobile- and web-based interventions for promoting healthy diets, preventing obesity, and improving health behaviors in children and adolescents: systematic review of randomized controlled trials. J Med Internet Res.

[7] Odendaal WA, Anstey Watkins J, Leon N, Goudge J, Griffiths F, Tomlinson M, Daniels K (2020). Health workers' perceptions and experiences of using mHealth technologies to deliver primary healthcare services: a qualitative evidence synthesis. Cochrane Database Syst Rev.

[8] Ferretti A, Hubbs S, Vayena E (2023). Global youth perspectives on digital health promotion: a scoping review. BMC Digit Health.

[9] Peuters C, DeSmet A, Maenhout L, Cardon G, Debeer D, Crombez G (2025). Adolescent engagement with a multicomponent mhealth tool: identifying usage patterns, determinants, and health behavior change in an intervention trial. JMIR Mhealth Uhealth.

[10] Eaton C, Vallejo N, McDonald X, Wu J, Rodríguez R, Muthusamy N, Mathioudakis N, Riekert KA (2024). User engagement with mhealth interventions to promote treatment adherence and self-management in people with chronic health conditions: systematic review. J Med Internet Res.

[11] O'Brien HL, Roll I, Kampen A, Davoudi N (2022). Rethinking (Dis)engagement in human-computer interaction. Comput Hum Behav.

[12] Nahum-Shani I, Yoon C (2024). Towards the science of engagement with digital interventions. Curr Dir Psychol Sci.

[13] Whitehead L, Robinson S, Arabiat D, Jenkins M, Morelius E (2024). The report of access and engagement with digital health interventions among children and young people: systematic review. JMIR Pediatr Parent.

[14] Psihogios AM, King-Dowling S, O'Hagan B, Darabos K, Maurer L, Young J, Fleisher L, Barakat LP, Szalda D, Hill-Kayser CE, Schwartz LA (2021). Contextual predictors of engagement in a tailored mHealth intervention for adolescent and young adult cancer survivors. Ann Behav Med.

[15] Li Y, Guo Y, Hong YA, Zeng Y, Monroe-Wise A, Zeng C, Zhu M, Zhang H, Qiao J, Xu Z, Cai W, Li L, Liu C (2022). Dose-response effects of patient engagement on health outcomes in an mHealth intervention: secondary analysis of a randomized controlled trial. JMIR Mhealth Uhealth.

[16] Yardley L, Spring BJ, Riper H, Morrison LG, Crane DH, Curtis K, Merchant GC, Naughton F, Blandford A (2016). Understanding and promoting effective engagement with digital behavior change interventions. Am J Prev Med.

[17] Jakob R, Harperink S, Rudolf AM, Fleisch E, Haug S, Mair JL, Salamanca-Sanabria A, Kowatsch T (2022). Factors Influencing Adherence to mHealth Apps for Prevention or Management of Noncommunicable Diseases: Systematic Review. J Med Internet Res.

[18] Duan Y, Shang B, Liang W, Du G, Yang M, Rhodes RE (2021). Effects of eHealth-based multiple health behavior change interventions on physical activity, healthy diet, and weight in people with noncommunicable diseases: systematic review and meta-analysis. J Med Internet Res.

[19] Szinay D, Jones A, Chadborn T, Brown J, Naughton F (2020). Influences on the uptake of and engagement with health and well-being smartphone apps: systematic review. J Med Internet Res.

[20] Perski O, Blandford A, West R, Michie S (2017). Conceptualising engagement with digital behaviour change interventions: a systematic review using principles from critical interpretive synthesis. Transl Behav Med.

[21] Schwarz A, Winkens LHH, de Vet E, Ossendrijver D, Bouwsema K, Simons M (2023). Design features associated with engagement in mobile health physical activity interventions among youth: systematic review of qualitative and quantitative studies. JMIR Mhealth Uhealth.

[22] Ghosh P, Proffitt R, Bosworth KT, Koopman RJ, Flowers L, Wilson G, Tosh AK, Braddock AS (2024). mHealth app features that facilitate adolescent use for lifestyle management, and are endorsed by caregivers and health care providers. Mhealth.

[23] Thornton L, Corliss C, Deen H, Teesson M, Champion KE, Partridge SR, Heinsch M, Spring B, Gardner LA, Rickwood D, Sunderland M, Newton NC, Zaman S, Redfern J, Osman B, Wilson J, Watt M, Kay-Lambkin F (2024). The triple E project: a factorial randomised controlled trial to enhance engagement with eHealth approaches to improve health risk behaviours among adolescents. BMC Public Health.

[24] Bendtsen M, Seiterö A, Bendtsen P, Henriksson H, Henriksson P, Thomas K, Löf M, Müssener U (2021). mHealth intervention for multiple lifestyle behaviour change among high school students in Sweden (LIFE4YOUth): protocol for a randomised controlled trial. BMC Public Health.

[25] Creswell JW, Plano Clark VL (2017). Designing and Conducting Mixed Methods Research.

[26] Kahwati L, Kane H (2020). Qualitative Comparative Analysis in Mixed Methods Research and Evaluation.

[27] Patton MQ (2015). Qualitative Research and Evaluation Methods - Integrating Theory and Practice.

[28] Tong A, Sainsbury P, Craig J (2007). Consolidated criteria for reporting qualitative research (COREQ): a 32-item checklist for interviews and focus groups. Int J Qual Health Care.

[29] O'Cathain A, Murphy E, Nicholl J (2008). The quality of mixed methods studies in health services research. J Health Serv Res Policy.

[30] Bendtsen M, Bendtsen P, Henriksson H, Henriksson P, Müssener U, Thomas K, Löf M (2020). The mobile health multiple lifestyle behavior interventions across the lifespan (MoBILE) research program: protocol for development, evaluation, and implementation. JMIR Res Protoc.

[31] Seiterö A, Henriksson P, Thomas K, Henriksson H, Löf M, Bendtsen M, Müssener U (2025). Effectiveness of a mobile phone-delivered multiple health behavior change intervention (LIFE4YOUth) in adolescents: randomized controlled trial. J Med Internet Res.

[32] Müssener U, Thomas K, Linderoth C, Löf M, Åsberg K, Henriksson P, Bendtsen M (2020). Development of an intervention targeting multiple health behaviors among high school students: participatory design study using heuristic evaluation and usability testing. JMIR Mhealth Uhealth.

[33] National guidelines: unhealthy lifestyle habits. Socialstyrelsen (the National Board of Health and Welfare in Sweden).

[34] Bendtsen M, Bendtsen P, Müssener U (2021). Six-month outcomes from the nexit junior trial of a text messaging smoking cessation intervention for high school students: randomized controlled trial with Bayesian analysis. JMIR Mhealth Uhealth.

[35] Thomas K, Müssener U, Linderoth C, Karlsson N, Bendtsen P, Bendtsen M (2018). Effectiveness of a text messaging-based intervention targeting alcohol consumption among university students: randomized controlled trial. JMIR Mhealth Uhealth.

[36] Ajzen I (1991). The theory of planned behavior. Organ Behav Hum Decis Process.

[37] Bandura A (2001). Social cognitive theory: an agentic perspective. Annu Rev Psychol.

[38] (2017). How a web session is defined in universal analytics. Analytics Help.

[39] Hedin L, Seiterö A, Crawford J, Bendtsen M, Löf M (2025). Mediated effects of LIFE4YOUth-a mobile health intervention for multiple lifestyle behavior change among high school students in Sweden: findings from a randomized controlled trial. BMC Public Health.

[40] Mahon C, Howard E, O'Reilly A, Dooley B, Fitzgerald A (2022). A cluster analysis of health behaviours and their relationship to mental health difficulties, life satisfaction and functioning in adolescents. Prev Med.

[41] McGovern CM, Militello LK, Arcoleo KJ, Melnyk BM (2018). Factors associated with healthy lifestyle behaviors among adolescents. J Pediatr Health Care.

[42] Mazur J, Szkultecka-Dębek M, Dzielska A, Drozd M, Małkowska-Szkutnik A (2018). What does the cantril ladder measure in adolescence?. Arch Med Sci.

[43] Rohlfing I (2019). The choice between crisp and fuzzy sets in qualitative comparative analysis and the ambiguous consequences for finding consistent set relations. Field Methods.

[44] Oana IE, Schneider CQ, Thomann E (2021). Qualitative Comparative Analysis with R: A Beginner's Guide.

[45] Thomann E, Ege J, Paustyan E (2022). Approaches to qualitative comparative analysis and good practices: a systematic review. Swiss Polit Sci Rev.

[46] Elgin DJ, Erickson E, Crews M, Kahwati LC, Kane HL (2024). Applying qualitative comparative analysis in large-N studies: a scoping review of good practices before, during, and after the analytic moment. Qual Quant.

[47] Schneider CQ, Wagemann C (2012). Doing justice to logical remainders in QCA: moving beyond the standard analysis. Disabil Rehabil.

[48] Oana IE, Schneider CQ (2021). A robustness test protocol for applied QCA: theory and R software application. Sociol Methods Res.

[49] Saarijärvi M, Wallin L, Moons P, Gyllensten H, Bratt EL (2020). Factors affecting adolescents' participation in randomized controlled trials evaluating the effectiveness of healthcare interventions: the case of the STEPSTONES project. BMC Med Res Methodol.

[50] Crutzen R, Ruiter R (2015). Interest in behaviour change interventions: a conceptual model. Eur Heal Psychol.

[51] Thornton L, Brown HM, Osman B, Stewart C, Whife J, Champion KE, Gardner LA, McBride N, Allsop S, Spring B, Teesson M (2022). Factors associated with adolescents’ engagement with a healthy lifestyles app. Procedia Comput Sci.

[52] Peuters C, Maenhout L, Cardon G, De Paepe A, DeSmet A, Lauwerier E, Leta K, Crombez G (2024). A mobile healthy lifestyle intervention to promote mental health in adolescence: a mixed-methods evaluation. BMC Public Health.

[53] Paz Castro R, Haug S, Filler A, Kowatsch T, Schaub MP (2017). Engagement within a mobile phone-based smoking cessation intervention for adolescents and its association with participant characteristics and outcomes. J Med Internet Res.

[54] Maenhout L, Peuters C, Cardon G, Crombez G, DeSmet A, Compernolle S (2022). Nonusage attrition of adolescents in an mHealth promotion intervention and the role of socioeconomic status: secondary analysis of a 2-arm cluster-controlled trial. JMIR Mhealth Uhealth.

[55] Melo GLR, Santo RE, Mas Clavel E, Bosque Prous M, Koehler K, Vidal-Alaball J, van der Waerden J, Gobiņa I, López-Gil JF, Lima R, Agostinis-Sobrinho C (2025). Digital dietary interventions for healthy adolescents: a systematic review of behavior change techniques, engagement strategies, and adherence. Clin Nutr.

[56] Whitaker V, Oldham M, Boyd J, Fairbrother H, Curtis P, Meier P, Holmes J (2021). Clustering of health-related behaviours within children aged 11-16: a systematic review. BMC Public Health.

[57] Noar SM, Chabot M, Zimmerman RS (2008). Applying health behavior theory to multiple behavior change: considerations and approaches. Prev Med.

[58] Moser A, Korstjens I (2018). Series: practical guidance to qualitative research. Part 3: sampling, data collection and analysis. Eur J Gen Pract.

[59] Barned C, Dobson J, Stintzi A, Mack D, O'Doherty KC (2018). Children's perspectives on the benefits and burdens of research participation. AJOB Empir Bioeth.

