# Supplementary material for: Adolescents’ Engagement With an mHealth Multiple Health Behavior Change Intervention (LIFE4YOUth): Mixed Methods and Qualitative Comparative Analysis
Source: JMIR Mhealth Uhealth. 2026 Mar 12;14:e88054. doi: 10.2196/88054 (PMC13022553; doi:10.2196/88054)
Supplement: Multimedia Appendix 1 [file mhealth_v14i1e88054_app1.docx]

# Complementary analyses of engagement patterns, including the validity of the identified clusters, the influence of the COVID-19 pandemic on engagement, and the robustness of the results.

Table of contents

[**Multimedia Appendix:** Adolescents’ Engagement with an mHealth Multiple Health Behavior Change Intervention (LIFE4YOUth): A Mixed-Methods and Qualitative Comparative Analysis 1](#_Toc221000072)

[Complementary analysis of engagement patterns 2](#_Toc221000073)

[Assessing the validity of the engagement clusters 2](#_Toc221000074)

[Influence on engagement of the COVID-19 pandemic 5](#_Toc221000075)

[QCA solution models 5](#_Toc221000076)

[Robustness tests 5](#_Toc221000077)

[Fit-oriented robustness test 5](#_Toc221000078)

[Case-oriented robustness test 6](#_Toc221000079)

[Variable-oriented robustness test 7](#_Toc221000080)

[Alternative solution models 8](#_Toc221000081)

**Table S1.** Baseline participant characteristics per engagement cluster.

**Table S2.** Fit statistics of the 2-, 3-, and 4-cluster solutions.

**Table S3.** Differences in engagement during and after COVID-19 based on two-sample t-tests

**Table S4.** Results of the robustness test of the most parsimonious solution.

**Table S5.** Associations between behavioral and psychosocial variables and engagement status from multinomial logistic regression models.

**Table S6.** Truth table for the negated outcome (not low engagement).

**Box S1**: The conservative and intermediate solution models for low engagement.

**Box S2**: The conservative, parsimonious, and intermediate solution models for the negated outcome (not low engagement).

**Figure S1.** Illustration of the proportion of typical and deviant cases with respect to their robustness.

### Complementary analysis of engagement patterns

#### Assessing the validity of the engagement clusters

**Table S1** presents an overview of the characteristics of participants at baseline specified per engagement cluster. K-means clustering was used to identify cut-offs for separating low-, medium-, and high-engagement groups within this dataset. Average silhouette width and the Adjusted Rand Index (ARI) were calculated to assess the validity of the selected 3-cluster solution, in comparison with the 2- and 4-cluster solutions. The silhouette values (**Table S2**) indicate that all solutions yielded well-separated and stable groupings based on the number of weeks participants completed the weekly screening questionnaire and actively engaged with dashboard content. However, the 4-cluster solution resulted in smaller and less stable clusters, as indicated by a lower ARI. The low-engagement cluster, as defined in this study (3-cluster solution), included participants who belonged to cluster 1 in at least one of the two engagement measures and had not activated the text-message program. The high-engagement cluster, as defined in this study (3-cluster solution), included participants who belonged to cluster 3 in at least one of the two engagement measures or had activated at least one text-message program.

| **Table S1.** Baseline participant characteristics per engagement cluster. | | | | |
| --- | --- | --- | --- | --- |
| Participant characteristics | Engagement | | | |
|  | Total  (n = 377) | Low  (n = 253) | Medium  (n = 57) | High  (n=67) |
| **Age, mean (SD)** | 17.18 (1.99) | 17.19 (1.19) | 16.93 (1.24) | 17.34 (1.20) |
| **Sex, n (%)** | | | | |
| Female | 265 (70) | 179 (71) | 33 (58) | 53 (79) |
| Male | 112 (30) | 74 (29) | 24 (42) | 14 (21) |
| **Parents’ education^1^, n (%)** | | | | |
| Primary | 25 (7) | 17 (7) | 3 (5) | 5 (7) |
| Secondary | 131 (35) | 90 (36) | 16 (28) | 25 (37) |
| Tertiary | 221 (59) | 146 (56) | 38 (67) | 37 (55) |
| **Economy^2^, n (%)** |  |  |  |  |
| Very good | 112 (30) | 76 (30) | 17 (30) | 19 (28) |
| Average | 232 (62) | 158 (62) | 34 (60) | 40 (60) |
| Not so good | 30 (8) | 16 (6) | 6 (11) | 8 (12) |
| Not good at all | 3 (1) | 3 (1) | 0 (0) | 0 (0) |
| **Region of birth^3^, n (%)** |  |  |  |  |
| Sweden | 324 (86) | 219 (87) | 50 (88) | 55 (82) |
| Other | 53 (14) | 34 (13) | 7 (12) | 12 (18) |
| **Parents’ region of birth^3^, n (%)** | | | | |
| Sweden | 268 (71) | 181 (72) | 43 (75) | 44 (66) |
| Other | 109 (29) | 72 (28) | 14 (25) | 23 (34) |
| **Satisfaction with life^4^, mean (SD)** | 6.83 (1.99) | 6.91 (1.98) | 6.44 (2.15) | 6.87 (1.91) |
| **Psychosocial variables^5^, mean (SD)** | | | | |
| Importance | 6.66 (2.53) | 6.45 (2.58) | 6.47 (2.61) | 7.58 (2.07) |
| Knowledge | 6.65 (2.31) | 6.99 (2.32) | 6.63 (2.43) | 6.55 (2.15) |
| Confidence | 6.24 (2.52) | 6.17 (2.54) | 5.65 (2.62) | 7.00 (2.18) |
| **Behavioral variables^6^, mean (SD)** | | | | |
| Weekly time spent in MVPA (minutes) | 323 (316) | 321(310) | 346 (360) | 309 (302) |
| Number of daily portions (100 g) of fruit and vegetables consumed | 1.43 (1.14) | 1.37 (1.08) | 1.30 (1.10) | 1.78 (1.34) |
| Number of weekly sugary drinks (33 cl) consumed | 3.27 (4.45) | 3.53 (4.75) | 3.44 (4.51) | 2.16 (2.83) |
| Number of weekly portions of candy or snacks consumed | 4.60 (5.55) | 4.29 (5.19) | 4.49 (5.62) | 5.88 (6.62) |
| Monthly frequency of heavy episodic drinking (ie., ≥ 4 standard drinks) | 1.21 (2.86) | 1.47 (3.35) | 0.93 (1.49) | 0.49 (0.91) |
| Number of weekly standard drinks (12 g pure alcohol) consumed | 1.75 (4.10) | 2.03 (4.49) | 1.75 (3.78) | 0.69 (2.31) |
| Smoking any cigarette in the past week, n (%) | 68 (18) | 53 (21) | 7 (12) | 8 (12) |

^1^ Assessed as “Please select the highest education for your mother and father”

^2^Assessed as “How would you describe the economic situation in your family?”

^3^Assessed as “Where were you/your parents born?”

^4^ Cantril’s Ladder [42]:“Thinking about your own life and personal circumstances, how satisfied are you with your life as a whole?”. Responses were given on an 11-point Likert scale where 0 represents not at all satisfied.

^5^ Defined as follows: Importance: “How important do you think it is to improve your lifestyle or sustain your healthy behaviors?” Confidence: “How confident are you that you will be able to change your lifestyle or sustain your healthy behaviors?”, Knowledge: “How well do you know how to change your lifestyle?”. Responses were provided on a 10-point Likert scale.

^6^ MVPA = Moderate to vigorous physical activity.

**Table S2.** Fit statistics of the 2-, 3-, and 4-cluster solutions.

| **Measure** | **Cluster** | **2 clusters**  **(n, mean)** | **3 clusters**  **(n, mean)** | **4 clusters**  **(n, mean)** |
| --- | --- | --- | --- | --- |
| **Weekly screenings** | Cluster 1 | 326 (1.05) | 278 (0.59) | 235 (0.33) |
|  | Cluster 2 | 51 (10.26) | 68 (4.59) | 82 (2.68) |
|  | Cluster 3 | - | 31 (12.58) | 36 (6.72) |
|  | Cluster 4 | - | - | 24 (13.58) |
| **Silhouette** |  | 0.789 | 0.722 | 0.712 |
| **Session episodes** |  |  |  |  |
|  | Cluster 1 | 335 (1.39) | 288 (0.75) | 264 (0.55) |
|  | Cluster 2 | 42 (15.00) | 57 (6.07) | 71 (4.51) |
|  | Cluster 3 | - | 32 (16.63) | 29 (12.41) |
|  | Cluster 4 | - | - | 13 (20.77) |
| **Silhouette** |  | 0.818 | 0.725 | 0.715 |
| **Adjusted Rand Index** |  | 0.775 | 0.784 | 0.674 |

#### Influence on engagement of the COVID-19 pandemic

To assess whether engagement differed during and after the COVID-19 pandemic, a complementary analysis was conducted using July 1, 2022, as the cutoff, corresponding to the transition between academic years. This date was selected because most Swedish high schools transitioned from remote to classroom-based teaching around the end of the school year. Engagement measures specified per study period are presented in **Table S3**.

**Table S3.** Differences in engagement during and after COVID-19 based on two-sample t-tests.

|  | **During COVID-19**  **n=103** | | **After COVID- 19**  **n=274** | **95% CI** | **p** |
| --- | --- | --- | --- | --- | --- |
| **Engagement measures^2^, mean (SD)** | | | | | |
| **Text-message programs** | | 0.18 (0.53) | 0.18 (0.54) | (-0.12, 0.12) | .99 |
| **Weekly screenings** | | 2.38 (4.09) | 2.26 (3.45) | (-0.71, 0.94) | .78 |
| **Session episodes** | | 3.11 (5.68) | 2.83 (4.56) | (-0.83, 1.39) | .62 |

^2^ Defined as number of activated text-message programs; number of completed weekly screenings; and number of session episodes with the dashboard content.

### QCA solution models

#### Robustness tests

##### Fit-oriented robustness test

By comparing the original most parsimonious solution model for low engagement with the model obtained after applying the alternative decisions (consistency threshold of 0.8 instead of 0.75; not excluding participants with activated text-message programs in the low engagement outcome; and using 5 as the frequency threshold instead of 1), we found that the part of the original solution that withstood all tested changes had a consistency of 0.95 and a coverage of 0.51 (**Table S4**). This indicates that the solution is highly stable with regard to consistency; however, the proportion of the outcome explained by the solution decreased, as only 51% of the original coverage remained across all tested changes. Furthermore, 48% and 97% of the original solution overlapped with the minTS and maxTS, respectively. This suggests that about half of the original solution is stable within the area where all possible solutions are coherent (minTS). In addition, almost the entire original solution is compatible with at least one of the possible solutions obtained when manipulating the parameters (maxTS).

**Table S4.** Results of the robustness test of the most parsimonious solution.

| **Sensitivity range** | | | | | | | |
| --- | --- | --- | --- | --- | --- | --- | --- |
| **Calibration anchors** | Condition | | Crossover point | | | | |
|  | IMP | | Lower bound: 7 | | Threshold: 7 | | Upper bound: 7 |
|  | LS | | Lower bound: 6 | | Threshold: 6 | | Upper bound: 6 |
|  | KNOW | | Lower bound: 7 | | Threshold: 7 | | Upper bound: 7 |
| **Parameters** | Raw consistency | | Lower: 0.75 | | Threshold: 0.75 | | Upper: 0.75 |
|  | Frequency | | Lower: 1 | | Threshold: 1 | | Upper: 2 |
| **Robustness parameters** | | | | | | | |
| **Fit oriented** | RF_cons_: 0.952 | RF_cov_: 0.508 | | RF_SC_minTS_: 0.483 | | RF_SC_maxTS_: 0.974 | |
| **Case oriented** | RCR_typ_: 0.508 | RCR_dev_: 0.379 | | RCC_Rank: 3 | | |  |
| **Worst performing model** | | | | | | | |
| ~HPB*HRB*~IMP*~KNOW + ~LS*HPB*HRB*IMP*KNOW + LS*HPB*HRB*IMP*~KNOW, obtained from changing the consistency threshold to 0.8. | | | | | | | |

RF = Robustness fit; RFcons = quantify how consistent the solution model is towards all tested changes; Rfcov = quantify how much of the solution coverage that remains towards all tested changes; RFSC_minTS = quantify the coincide between the solution model and the area where all possible solutions agree; RFSC_maxTS = quantify the coincide between the solution model and the entire area of possible solutions; RCR = Robustness case ratio, RCRtyp = quantify the proportion of robust typical cases; RCRdev = quantify the proportion of [robust] deviant cases; RCC_Rank = range from 1 to 4 where 1 indicates the most robust scenario.

##### Case-oriented robustness test

**Figure S1** shows the results of the case-oriented robustness test. Of all low-engaged participants covered by the original most parsimonious solution, 25% were also covered by the minTS (i.e., robust typical cases), highlighting one configuration that was stable across the tested changes (~HPB*HRB*~IMP*~KNOW). Almost as many participants (24%) were not covered by the minTS, highlighting 4 configurations that were less stable when the consistency threshold was changed (HPB*IMP*~LS + HPB*HRB*~KNOW*LS + HRB*IMP*~KNOW*LS + ~HPB*HRB*~IMP*KNOW*~LS). Altogether, the following characteristics remained stable toward all changes tested, indicating a strong association with the outcome low engagement: not being engaged in health-promoting behaviors but being engaged in health-risk behaviors and not considering healthy behaviors as very important and not being very skilled in how to change behaviors.

**
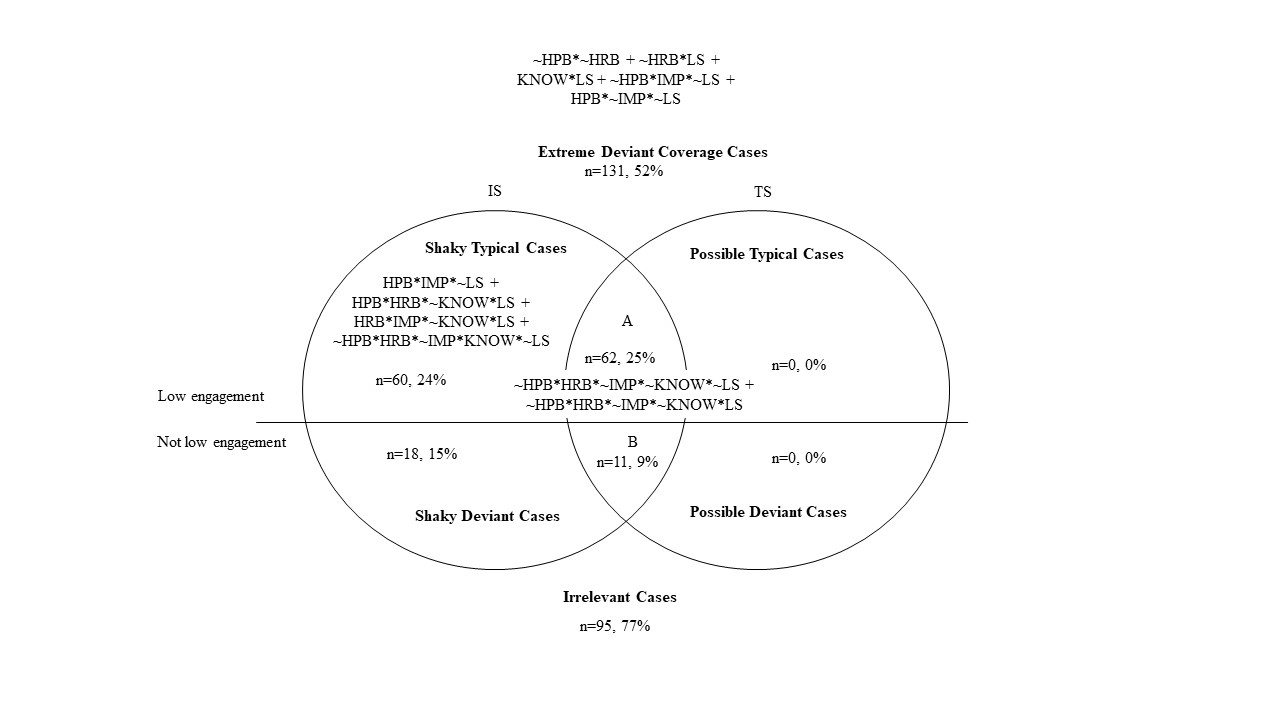
Figure S1.** Illustration of the proportion of typical and deviant cases with respect to their robustness.

##### Variable-oriented robustness test

Regression analyses were conducted to complement the QCA findings regarding associations between behavioral and psychosocial variables (i.e., conditions). **Table S5** presents the results of regression models used to examine associations between engagement and each explanatory variable in isolation, as well as models that included all variables simultaneously. Engagement in health-risk behaviors (alcohol consumption or cigarette smoking) was associated with increased odds of being low-engaged compared with high-engaged (OR = 2.078, 95% CI 1.176–3.672, p = .012). In addition, participants who considered healthy behaviors to be very important were less likely to be low-engaged (OR = 0.532, 95% CI 0.299–0.485) or medium-engaged compared with high-engaged (OR = 0.359, 95% CI 0.168–0.767).

| **Table S5.** Associations between behavioral and psychosocial variables and engagement status from multinomial logistic regression models.  **Table ?? Association between explanatory variables and engagement status from multinomial logistic regression models.** | | | | | | |
| --- | --- | --- | --- | --- | --- | --- |
|  | **Univariate analysis^1^**  **n=377** | | | **Multivariable analysis^2^**  **n=377** | | |
|  | **OR^3^** | **95% CI** | **p** | **OR^3^** | **95% CI** | **p** |
| **Low engagement** |  |  |  |  |  |  |
| **HPB** | 0.732 | (0.412, 1.300) | 0.287 | 0.810 | (0.449, 1.463) | 0.484 |
| **HRB** | **2.329** | **(1.333, 4.069)** | **0.003** | **2.078** | **(1.176, 3.672)** | **0.012** |
| **LS** | 0.825 | (0.435, 1.562) | 0.554 | 0.880 | (0.454, 1.706) | 0.705 |
| **IMP** | **0.449** | **(0.259, 0.777)** | **0.004** | **0.532** | **(0.299, 0.946)** | **0.032** |
| **KNOW** | 0.791 | (0.446, 1.402) | 0.422 | 0.885 | (0.485, 1.614) | 0.690 |
| **Medium engagement** | | | | | |  |
| **HPB** | 0.683 | (0.315, 1.484) | 0.336 | 0.764 | (0.346, 1.685) | 0.504 |
| **HRB** | 1.303 | (0.631, 2.691) | 0.474 | 1.115 | (0.531, 2.340) | 0.774 |
| **LS** | 1.355 | (0.610, 3.010) | 0.456 | 1.636 | (0.714, 3.749) | 0.245 |
| **IMP** | **0.394** | **(0.190, 0.814)** | **0.012** | **0.359** | **(0.168, 0.767)** | **0.008** |
| **KNOW** | 1.294 | (0.623, 2.688) | 0.489 | 1.704 | (0.789, 3.678) | 0.175 |

^1^Univariate analyses assessed each explanatory variable.
^2^Multivariable analyses included all explanatory variables simultaneously, which estimates each association while holding the others constant.

^3^Reference category in multinomial logistic models is “high engagement”.

Abbreviations: HPB = Engaged in health protective behaviors (sufficient physical activity or healthy diet) (1/0); HRB = Engaged in health-risk behaviors (alcohol consumption or cigarette smoking) (1/0); LS = Dissatisfaction with life (1/0); IMP = Considering healthy behaviors as very important (1/0); KNOW = High-skilled in how-to change behaviors (1/0).

#### Alternative solution models

**Box 1** shows the conservative and intermediate solution models obtained from the primary analysis of the low engagement outcome. The analysis of the negated outcome was conducted to ensure that the configurations associated with low engagement were not simultaneously associated with higher levels of engagement. **Table S6** presents the truth table used for the minimization process of the not-low-engagement outcome, and **Box 2** shows the three solution models resulting from this process. These results further support that the parsimonious solution model was stable. Moreover, the conditions included in the QCA were more relevant for explaining low engagement than for explaining not low engagement.

**Box 1.** The conservative and intermediate solution models for **low engagement**.

**Conservative solution (low engagement):**

M1: ~LS*HPB*IMP + LS*HRB*~KNOW + ~LS*~HPB*HRB*~IMP -> LE

inclS PRI covS covU

------------------------------------------------

1 ~LS*HPB*IMP 0.762 0.762 0.126 0.126

2 LS*HRB*~KNOW 0.828 0.828 0.095 0.095

3 ~LS*~HPB*HRB*~IMP 0.825 0.825 0.261 0.261

------------------------------------------------

**M1** 0.808 0.808 0.482

**Intermediate solution (low engagement):**

Directed expectations: "HRB, ~HPB, LS, ~KNOW, ~IMP

M1: ~LS*HPB*IMP + LS*HRB*~KNOW + ~LS*~HPB*HRB*~IMP -> LE

inclS PRI covS covU

------------------------------------------------

1 ~LS*HPB*IMP 0.762 0.762 0.126 0.126

2 LS*HRB*~KNOW 0.828 0.828 0.095 0.095

3 ~LS*~HPB*HRB*~IMP 0.825 0.825 0.261 0.261

------------------------------------------------

**M1** 0.808 0.808 0.482

**Table S6.** Truth table for the negated outcome (**not low engagement**)

| **#** | **LS** | **HPB** | **HRB** | **IMP** | **KNOW** | **OUT** | **n** | **Incl** |
| --- | --- | --- | --- | --- | --- | --- | --- | --- |
| 18 | 1 | 0 | 0 | 0 | 1 | **1** | 2 | 1.000 |
| 27 | 1 | 1 | 0 | 1 | 0 | **1** | 6 | 1.000 |
| 22 | 1 | 0 | 1 | 0 | 1 | **0** | 3 | 0.667 |
| 14 | 0 | 1 | 1 | 0 | 1 | **0** | 5 | 0.600 |
| 2 | 0 | 0 | 0 | 0 | 1 | **0** | 12 | 0.500 |
| 3 | 0 | 0 | 0 | 1 | 0 | **0** | 24 | 0.500 |
| 24 | 1 | 0 | 1 | 1 | 1 | **0** | 2 | 0.500 |
| 4 | 0 | 0 | 0 | 1 | 1 | **0** | 20 | 0.450 |
| 9 | 0 | 1 | 0 | 0 | 0 | **0** | 9 | 0.444 |
| 8 | 0 | 0 | 1 | 1 | 1 | **0** | 16 | 0.438 |
| 19 | 1 | 0 | 0 | 1 | 0 | **0** | 12 | 0.417 |
| 17 | 1 | 0 | 0 | 0 | 0 | **0** | 15 | 0.400 |
| 20 | 1 | 0 | 0 | 1 | 1 | **0** | 5 | 0.400 |
| 25 | 1 | 1 | 0 | 0 | 0 | **0** | 5 | 0.400 |
| 10 | 0 | 1 | 0 | 0 | 1 | **0** | 8 | 0.375 |
| 32 | 1 | 1 | 1 | 1 | 1 | **0** | 6 | 0.333 |
| 13 | 0 | 1 | 1 | 0 | 0 | **0** | 22 | 0.318 |
| 1 | 0 | 0 | 0 | 0 | 0 | **0** | 39 | 0.308 |
| 7 | 0 | 0 | 1 | 1 | 0 | **0** | 15 | 0.267 |
| 11 | 0 | 1 | 0 | 1 | 0 | **0** | 16 | 0.250 |
| 15 | 0 | 1 | 1 | 1 | 0 | **0** | 8 | 0.270 |
| 29 | 1 | 1 | 1 | 0 | 0 | **0** | 4 | 0.250 |
| 12 | 0 | 1 | 0 | 1 | 1 | **0** | 13 | 0.231 |
| 23 | 1 | 0 | 1 | 1 | 0 | **0** | 13 | 0.231 |
| 6 | 0 | 0 | 1 | 0 | 0 | **0** | 23 | 0.217 |
| 16 | 0 | 1 | 1 | 1 | 1 | **0** | 5 | 0.200 |
| 5 | 0 | 0 | 1 | 0 | 0 | **0** | 57 | 0.158 |
| 21 | 1 | 0 | 1 | 0 | 0 | **0** | 11 | 0.091 |
| 31 | 1 | 1 | 1 | 1 | 0 | **0** | 1 | 0.000 |
| 26 | 1 | 1 | 0 | 0 | 1 | ? | 0 | - |
| 28 | 1 | 1 | 0 | 1 | 1 | ? | 0 | - |
| 30 | 1 | 1 | 1 | 0 | 1 | ? | 0 | - |

Configurations based on the presence (1) or absence (0) of the following conditions: LS: Being dissatisfied with life (≤5 on a 11-point Likert scale); HPB: Engagement in health-promoting behaviors (≥420 min MVPA or ≥3 daily portions (100g) of fruit and vegetables and <4 portions of candy or cakes or <2 cans (33 cl) of sugary drinks); HRB: Engagement in health-risk behaviors (smoking ≥1 cigarette or being <18 years old and drinking any alcohol or being ≥18 years old and having consumed ≥10 standard drinks (a´12 g pure alcohol) or being engaged in heavy episodic drinking (≥4 standard drinks on a single occasion) at least once in the past month); IMP: Considering healthy behaviors as very important (≥8 on a 10-point scale); KNOW: High-skilled in how-to change behaviors (≥8 on a 10-point scale); OUT: 1 = ≥75% of all participants within the configuration had medium- or high engagement, 0 = <75% had medium- or high engagement, ? = not represented in the data; n = number of participants represented by the configuration; Incl = quantifies the degree of a set relationship where 1 indicates a strong relationship between the configuration and the outcome.

**Box 2.** The conservative, parsimonious, and intermediate solution models for the negated outcome (**not low engagement).**

**Conservative solution (negated outcome):**

M1: LS*HPB*~HRB*IMP*~KNOW + LS*~HPB*~HRB*~IMP*KNOW -> ~LE

inclS PRI covS covU cases

-----------------------------------------------------------------------------

1 LS*HPB*~HRB*IMP*~KNOW 1.000 1.000 0.048 0.048

2 LS*~HPB*~HRB*~IMP*KNOW 1.000 1.000 0.016 0.016

-----------------------------------------------------------------------------

**M1** 1.000 1.000 0.065

**Parsimonious solution (negated outcome)**:

M1: LS*HPB*~HRB*IMP + LS*~HRB*~IMP*KNOW -> ~LE

inclS PRI covS covU cases

----------------------------------------------------------------------------

1 LS*HPB*~HRB*IMP 1.000 1.000 0.048 0.048

2 LS*~HRB*~IMP*KNOW 1.000 1.000 0.016 0.016

----------------------------------------------------------------------------

**M1** 1.000 1.000 0.065

**Intermediate solution (negated outcome)**:

Directed expectations: "~HRB, HPB, LS, ~KNOW, ~IMP"

M1: LS*~HRB*~IMP*KNOW + LS*HPB*~HRB*IMP*~KNOW -> ~LE

inclS PRI covS covU cases

----------------------------------------------------------------------------

1 LS*~HRB*~IMP*KNOW 1.000 1.000 0.016 0.016

2 LS*HPB*~HRB*IMP*~KNOW 1.000 1.000 0.048 0.048

----------------------------------------------------------------------------

**M1** 1.000 1.000 0.065
